# Effect of Ceftaroline, Ceftazidime/Avibactam, Ceftolozane/Tazobactam, and Meropenem/Vaborbactam on Establishment of Colonization by Vancomycin-Resistant Enterococci and *Klebsiella pneumoniae* in Mice

**DOI:** 10.20411/pai.v9i2.711

**Published:** 2024-09-24

**Authors:** Bryan S. Hausman, Samir Memic, Jennifer L. Cadnum, Elizabeth G. Zink, Brigid M. Wilson, Curtis J. Donskey

**Affiliations:** 1 Case Western Reserve University School of Medicine, Cleveland, Ohio; 2 Research Service, Louis Stokes Cleveland VA Medical Center, Cleveland, Ohio; 3 Geriatric Research, Education and Clinical Center, Louis Stokes Cleveland VA Medical Center, Cleveland, Ohio

**Keywords:** ceftaroline, ceftazidime/avibactam, ceftolozane/tazobactam, meropenem/vaborbactam, colonization resistance, vancomycin-resistant enterococci

## Abstract

**Background:**

The potential for promotion of intestinal colonization with healthcare-associated pathogens by new antibiotics used to treat infections due to multidrug-resistant Gram-negative bacilli is unclear.

**Methods:**

Mice treated for 3 days with daily subcutaneous phosphate-buffered saline (control), ceftazidime/avibactam, ceftolozane/tazobactam, ceftaroline, and meropenem/vaborbactam were challenged with 10,000 colony-forming units (CFU) of vancomycin-resistant *Enterococcus* (VRE) resistant to each of the antibioics or carbapenemase-producing *Klebsiella pneumoniae* 1 day after the final treatment dose. The concentrations of VRE or *K. pneumoniae* in stool were measured on days 1, 3, 6, and 15 after challenge.

**Results:**

Control mice had transient low levels of VRE or *K. pneumoniae* (<3 log_10_ CFU/g) detected in stool with negative cultures on days 6 and 15 after challenge. In comparison to control mice, each of the antibiotics promoted establishment of high-density colonization with VRE (mean concentration, >8 log_10_ CFU/g of stool on day 1 after challenge) that persisted at >4 log_10_ CFU/g of stool through day 15 (*P*<0.01). In comparison to control mice, meropenem/vaborbactam and ceftaroline promoted high-density colonization with *K. pneumoniae* (peak concentration, >8 log_10_ CFU/g of stool) (*P*<0.01), ceftolozane/tazobactam promoted colonization to a lesser degree (peak concentration, >5 log_10_ CFU/g of stool), and ceftazidime/avibactam did not promote colonization (*P*>0.05).

**Conclusions:**

Our results suggest that several beta-lactam antibiotics recently developed for treatment of infections with resistant Gram-negative bacilli have the potential to promote colonization by healthcare-associated pathogens. Additional studies are needed to examine the impact of these agents in patients.

## INTRODUCTION

The indigenous microbiota of the intestinal tract provide an important host defense, termed colonization resistance, by preventing colonization with potentially pathogenic microorganisms [1]. This defense is primarily mediated by anaerobic bacteria [1–3]. Antibiotics with activity against intestinal anaerobes that are excreted into the intestinal tract (eg, clindamycin, ceftriaxone) can disrupt colonization resistance, thereby promoting overgrowth of antibiotic-resistant bacteria, *Clostridioides difficile*, and *Candida* species [1–11]. After completion of treatment, recovery of the indigenous microbiota occurs over a period of days to weeks. Thus, susceptibility to overgrowth of pathogens persists during the period of recovery of the indigenous microbiota [1, 5, 9, 10]. For example, the risk of *C. difficile* infection may persist for several weeks after antibiotic treatment is discontinued [10]. In mice, antibiotics that do not alter intestinal anaerobes due to lack of antian-aerobic activity (eg, aztreonam, lolamicin) and/or minimal excretion into the intestinal tract (eg, cefepime) do not alter colonization resistance [1, 5, 12].

Some antibiotics have activity against pathogens and can inhibit colonization during the course of treatment if sufficient concentrations are achieved in the intestinal tract [1, 4–10]. Such antibiotics may have a biphasic effect on promotion of susceptible pathogens if they also disrupt the anaerobic microbiota (ie, inhibition of colonization during treatment due to inhibitory activity but promotion if exposure occurs after treatment during the vulnerable period when the indigenous microbiota is still recovering) [1, 5, 9, 10]. For example, piperacillin/tazobactam is excreted in bile and can inhibit establishment of colonization by susceptible Gram-negative bacilli, vancomycin-resistant enterococci (VRE), and *C. difficile* during treatment, but promote colonization by the same pathogens if exposure occurs after treatment prior to recovery of the indigenous micro-biota [1, 5, 9].

In recent years, several new antibiotics have become available for treatment of multidrug-resistant Gram-negative bacilli. The potential for many of these agents to disrupt the indigenous micro-biota of the colon and promote colonization by pathogens is unclear. Here, we used an established mouse model to examine the impact of ceftaroline, ceftazidime/avibactam, ceftolozane/tazobactam, and meropenem/vaborbactam on establishment of colonization by VRE and a carbapenemase-producing *K. pneumoniae* isolate. These agents are primarily renally excreted but based on studies with radiolabeled antibiotic are excreted to a variable degree in stool of healthy volunteers (ceftaroline, 6%; ceftazidime/avibactam, 0.2%; meropenem/vaborbactam, 2%) or rats (ceftolozane/tazobactam, 2.2%) [13–16]. Thus, these agents have the potential to alter colonization resistance. For ceftazidime/avibactam, there is evidence that colonization resistance might be altered as this agent suppressed anaerobes but promoted overgrowth of enterococci in stool of healthy volunteers [17].

## METHODS

### Mice

The Animal Care Committee of the Cleveland Veterans Affairs Medical Center approved the experimental protocol. Female CF-1 mice (4-8 per group) weighing 30-45g (Harlan Sprague-Dawley) were housed in individual cages with plastic filter tops to prevent cross-contamination among animals.

### The Pathogens Studied

*Klebsiella pneumoniae* VA367 is a thoroughly characterized clinical carbapenemase-producing isolate that was used in a previous mouse model study [18]. The isolate contains *bla*KPC-3, *bla*TEM-1, *bla*SHV-11, and *bla*SHV-12 as well as *qnrB19* and *aac(6)-lb* [18–20]. In addition, this isolate has disruptions in the coding sequences of outer membrane protein genes *ompK35*, *ompK36*, and *ompK37* [19, 20]. *Enterococcus faecium* C68 is a VanB-type clinical VRE isolate used in previous mouse model studies [21].

### Susceptibility Testing and Bioassay for Antibiotic Concentrations in Stool

Broth dilution minimum inhibitory concentrations (MICs) of the test antibiotics for *K. pneumoniae* VA367 and VRE C68 were determined using standard methods for susceptibility testing of aerobic and anaerobic bacteria [22]. For ceftazidime/avibactam, ceftolozane/tazobactam, and ceftaroline, MICs were determined using ETEST (bioMerieux). The concentration of the antibiotics in stool pellets was determined by an agar diffusion assay with *Escherichia coli* or *Bacillus subtilis* as the indicator strain [6]. The limit of detection was ∼2 μg/mL.

### Antibiotic Dose Selection

In a previous mouse model study, we found that a dose of 5-times the usual human dose of doxycyline on a mg/kg basis resulted in drug concentrations in stool that were similar to levels measured in human volunteers [8]. Therefore, we performed preliminary experiments to assess antibiotic concentrations in stool after doses equal to or 5-times the usual human dose. Mice (5/group) received subcutaneous treatment with the antibiotics used for treatment of Gram-negative bacilli once daily for 3 days in a dose equal to or 5-times the usual human dose on a mg/kg basis. Fecal pellets were collected at 4, 8, and 24 hours after the third dose, and antibiotic concentrations were measured by bioassay as described previously. In these experiments, 0 to 3 of 5 mice in each group receiving the usual human dose had detectable levels of antibiotic in stool, whereas 4 to 5 of 5 mice receiving 5-times the usual dose had detectable antibiotic in stool. The peak concentrations of ceftazidime/avibactam in stool of mice receiving 5-times the usual human dose were equivalent to levels previously reported to be present in stool of human volunteers after 2 days of treatment (ceftazidime: median 28, range 0-291 μg/g; avibactam: median 1, range 0-14 μg/g) [17]. Therefore, doses of 5-times the usual human dose were used for subsequent experiments.

The daily doses of the test antibiotics were ceftazidime/avibactam (18.75 mg; 625 mg/kg), ceftolozane/tazobactam (22.5 mg; 750 mg/kg), ceftaroline (4.5 mg; 150 mg/kg), and meropenem/vaborbactam (30 mg; 1,000 mg/kg). Clindamycin was included as a positive control that has previously been shown to promote colonization by VRE and *K. pneumoniae*; the dose of clindamycin was 1.2 mg (40 mg/kg) [1, 5].

### Effect of Antibiotic Treatment on Indigenous Facultative Gram-negative Bacilli and on Establishment of Colonization by VRE and *K. pneumoniae*

Initial experiments were conducted to examine the impact of treatment on indigenous facultative Gram-negative bacilli and on establishment of VRE colonization. Mice (7-8 per group) received once daily treatment for 3 days with subcutaneous phosphate-buffered saline (PBS) (0.1 mL), ceftazidime/avibactam, ceftolozane-tazobactam, ceftaroline, meropenem-vaborbactam, or clindamycin (positive control). The antibiotics were administered in 0.1 mL of PBS. After the third dose, stool pellets were collected to assess the impact of treatment on levels of facultative Gram-negative bacilli. Fresh stool specimens were processed as described elsewhere [5, 13]. To quantify facultative Gram-negative bacilli, diluted samples were plated onto MacConkey agar (Becton Dickinson). Plates were incubated for 24 hours at 37°C and the number of colony-forming units (CFU) of Gram-negative bacilli per gram of stool was calculated.

One day (24-28 hours) after the third antibiotic dose, the mice received 10,000 CFU of VRE C68 by orogastric gavage [4, 9, 18]. Fecal pellets were collected at 1, 3, 6, and 15 days after the VRE exposure. To quantify VRE C68, diluted samples were plated onto Enterococcosel agar (Becton Dickinson) containing 20 μg/mL vancomycin. Plates were incubated for 72 hours at 37°C and the CFU of VRE per gram of stool was calculated.

Similar experiments were conducted to examine the impact of treatment on establishment of colonization with *K. pneumoniae*. Twenty-four hours after the last antibiotic dose, the mice received 10,000 CFU of *K. pneumoniae* VA367 by orogastric gavage. To quantify *K. pneumoniae* VA367, diluted samples were plated onto MacConkey agar (Difco Laboratories) containing 0.5 μg/mL imipenem/cilastatin. Plates were incubated for 24 hours at 37°C and the number of CFU per gram of stool was calculated.

### Additional Experiments to Assess Meropenem/Vaborbactam Stool Concentrations and Impact on *K. pneumoniae* VA367 Colonization

The colonization experiments demonstrated that meropenem/vaborbactam-treated mice developed high-density colonization with meropenem/vaborbactam susceptible *K. pneumoniae* VA367 despite the initial experiments demonstrating substantial drug levels in stool of 4 of 5 mice at 24 hours. Due to concern that technical error might have led to inaccurate results, we performed 2 additional sets of experiments (12 mice total) to re-assess meropenem/vaborbactam drug levels in stool and impact on establishment of colonization by* K. pneumoniae* VA367 colonization.

### Data Analysis

For *K. pneumoniae* VA367, a linear mixed model was used to compare concentrations of organisms among the treatment groups. For VRE, a 2-way repeated measures analysis of variance (ANOVA) was used to compare groups; the sphericity assumption was violated (ie, the variances of the differences between all the combinations of related groups were not equal) and therefore the Greenhouse-Geisser method was used to apply a correction to the degrees of freedom used to calculate the F-ratio. For both organisms, Dunnett's method for multiple comparisons was used as a post-hoc test to compare the treatment groups against the saline controls for each day. Data analysis was performed in R Version 4.2.2.

## RESULTS

### Susceptibility Testing

Table 1 shows the MICs of the test antibiotics against *K. pneumoniae* VA367 and VRE C68. VRE C68 was resistant to all the test antibiotics. *K. pneumoniae* VA367 was resistant to ceftolozane/tazobactam and ceftaroline, but susceptible to ceftazidime/avibactam and meropenem/vaborbactam.

**Table 1.** Minimum Inhibitory Concentrations (MICs) of the Test Antibiotics Against the Challenge Organisms

Antibiotic	MIC (μg/mL)^a^	
	*Klebsiella pneumoniae*	VA367 VRE C68
Ceftazidime/avibactam	<0.016	(R) ^b^
Ceftolozane/tazobactam	24	(R)
Ceftaroline	96	(500)
Meropenem/vaborbactam	(<1)	(250)

a Antibiotic Susceptibility Testing was done with an E-test (values in parenthesis denote the agar dilution method was used)

b R: resistant

VRE, vancomycin-resistant *Enterococcus*

### Effect of Antibiotic Treatment on Indigenous Facultative Gram-negative Bacilli

All mice had ∼6 log_10_ CFU of facultative Gram-negative bacilli identified as *Escherichia coli* at baseline. After 3 days of antibiotic treatment, mice treated with ceftazidime/avibactam, ceftolozane/tazobactam, ceftaroline, and meropenem/vaborbactam had no detectable facultative Gram-negative bacilli (limit of detection, ∼1 log_10_ CFU/g stool), whereas the control mice and mice treated with clindamycin maintained ∼6 log_10_ CFU/g stool.

### Effect of Antibiotic Treatment on Establishment of Colonization by VRE and *K. pneumoniae*

**Figure 1.**
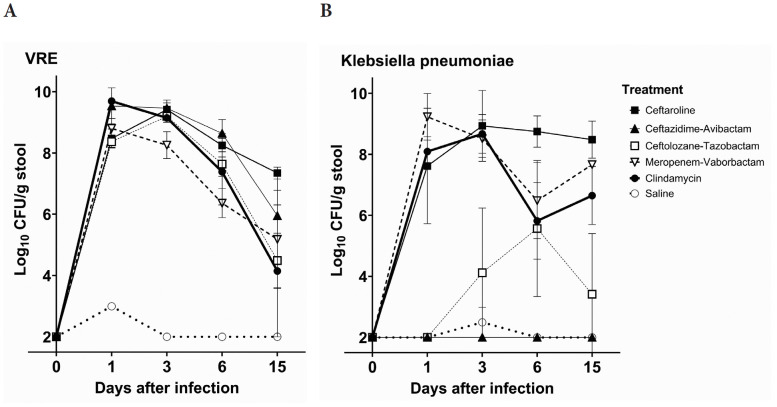
Mean +SE concentrations of vancomycin-resistant enterococci (VRE) (A) and *Klebsiella pneumoniae* VA367 (B) in stool of mice after orogastric gavage of 10,000 colony-forming units (CFU) of the test organisms 1 day after the final of 3 daily antibiotic doses. Error bars show the standard error.

Figure 1A shows the impact of antibiotic treatment on establishment of colonization with VRE C68. Control mice treated with PBS had only transient low levels of VRE (<3 log_10_ CFU/g) detected in stool on day 1 but no detectable VRE on days 3, 6, and 15. In comparison to the control mice, each of the antibiotics promoted establishment of high-density colonization with VRE (mean concentration, >8 log_10_ CFU/g of stool on day 1 after challenge) that persisted at >4 log_10_ CFU/g of stool through day 15 (*P*<0.01).

Figure 1B shows the impact of antibiotic treatment on establishment of colonization with *K. pneumoniae* VA367. Control mice had no detectable *K. pneumoniae* in stool with the exception of 1 mouse with transient low levels detected in stool on day 3. In comparison to the controls, meropenem/vaborbactam and ceftaroline promoted high-density colonization with *K. pneumoniae* (peak concentration, >8 log_10_ CFU/g of stool) (*P*<0.01), whereas ceftolozane/tazobactam promoted colonization to a lesser degree (peak concentration, >5 log_10_ CFU/g of stool). Ceftazidime/avibactam did not promote colonization (*P*>0.05).

### Additional Experiments to Assess Meropenem/Vaborbactam Stool Concentrations and Impact on *K. pneumoniae* VA367 Colonization

In contrast to the initial experiments, none of the 12 mice treated with meropenem/vaborbactam had detectable drug levels in stool collected 4, 8, and 24 hours after the third dose. All 8 meropenem/vaborbactam-treated mice that were challenged with *K. pneumoniae* VA367 developed high-density colonization (peak concentration, >8 log_10_ CFU/g of stool).

### Concentration of Antibiotics in Stool

**Figure 2.**
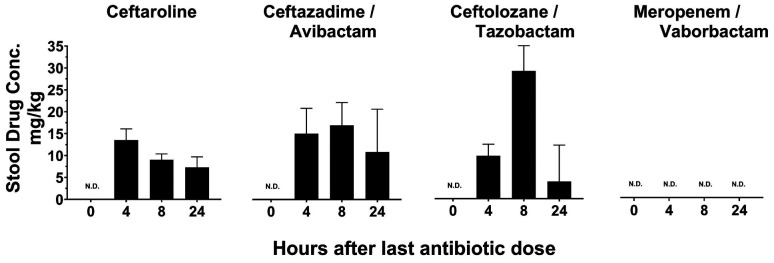
Concentration (mg/kg) of antibiotics in stool of mice at 4, 8, and 24 hours after the final of 3 daily antibiotic doses. N.D., not detected.

**Note.** Meropenem/vaborbactam was detected in stool of 4 of 5 mice up to 24 hours after the final dose in an initial experiment, but technical error was suspected. These results were excluded because, in 2 subsequent experiments, 12 of 12 mice had no detectable drug in stool. Conc., concentration. Error bars show standard error.

Figure 2 shows the concentrations of the test antibiotics in stool specimens collected 4, 8, and 24 hours after the third of 3 daily doses of the antibiotics at 5-times the usual human dose on a mg/kg basis. Treatment with ceftolozane/tazobactam, ceftaroline, and ceftazidime/avibactam resulted in detectable concentrations of the antibiotics in stool at 4, 8, and 24 hours after dosing. As noted previously, meropenem/vaborbactam was not detected in stool in 2 of the 3 experiments.

## DISCUSSION

Antibiotics vary in their potential to promote colonization by healthcare-associated pathogens [1, 5]. Thus, there is a need for evaluations of the impact of new antibiotics on the indigenous intestinal microbiota and on colonization resistance to healthcare-associated pathogens. In the current mouse model study, we found that ceftazidime/avibactam, ceftolozane/tazobactam, ceftaroline, and meropenem/vaborbactam suppressed indigenous Gram-negative bacilli. These antibiotics altered colonization resistance as evidenced by promotion of colonization by a VRE strain resistant to each agent.

The impact of the test antibiotics on colonization by *K. pneumoniae* VA367 highlights the fact that agents with activity against pathogens can inhibit the establishment of colonization during treatment. Ceftazidime/avibactam is excreted in the intestinal tract and has potent activity against *K. pneumoniae* VA367; this agent was detected in substantial concentrations in stool 1 day after the final dose (mean concentration, 4 mg/kg of stool) and prevented establishment of colonization by *K. pneumoniae* VA367 when challenge occurred 24 to 28 hours after the final dose. One limitation of our experimental model is that we did not challenge mice with *K. pneumoniae* at multiple time points after treatment. As noted previously, it should be appreciated that results can differ based on when exposure to pathogens occurs. Based on previous studies, it is plausible that ceftazidime/avibactam might exhibit a biphasic effect on colonization by *K. pneumoniae* VA367 (ie, suppression of colonization when challenge occurs during and soon after treatment while drug levels are present in the intestinal tract but promotion of colonization after drug levels have decreased to undetectable levels but the anaerobic microbiota has not fully recovered).

Meropenem/vaborbactam also has potent activity against *K. pneumoniae* VA367, but did not inhibit establishment of colonization. Although meropenem/vaborbactam was detected in stool during initial experiments, in 2 subsequent experiments it was not detectable in stool, including in an experiment in which 8 of 8 treated mice developed high-density colonization with VA367. Thus, it is likely that meropenem/vaborbactam did not prevent establishment of colonization with VA367 because the concentration of drug in the intestinal tract was below inhibitory levels when the challenge occurred (24-28 hours after the final dose). The finding that meropenem/vaborbactam was not detectable in stool is consistent with prior evidence that only 0.2% of radiolabeled meropenem/vaborbactam is excreted in stool of healthy volunteers [15]. Moreover, in a previous study in healthy human volunteers receiving intravenous meropenem, no drug was detected in stool by bioassay despite evidence of alteration of the aerobic and anaerobic microbiota [23]. In contrast to ceftazidime/avibactam and meropenem/vaborbactam, ceftolozane/tazobactam and ceftaroline have reduced activity against VA367 and promoted colonization with this organism.

Our findings for ceftazidime/avibactam are consistent with a previous study in 12 healthy human volunteers [17]. In that study, ceftazidime/avibactam treatment suppressed levels of *Escherichia coli*, lactobacilli, bifidobacteria, clostridia, and bacteroides, whereas enterococci increased, and 5 participants acquired *C. difficile* colonization [17]. However, our results differ from a similar previous study in which healthy human volunteers were treated with ceftaroline [24]. In that study, no detectable levels of ceftaroline were found in stool specimens and treatment had no significant ecological impact on the intestinal microbiota [24]. However, it is notable that 6% of radiolabeled ceftaroline was recovered in the stool of human volunteers [13]. Thus, additional studies are needed to assess the impact of ceftaroline on the microbiota of humans.

Our study has some limitations. We used a mouse model in which healthy mice were dosed once daily with the antibiotics. Antibiotic excretion in the intestinal tract of mice and humans may differ. The challenge with pathogens occurred once approximately 1 day after the final antibiotic dose. In clinical settings, it is anticipated that patients may be exposed to pathogens intermittently during and after antibiotic therapy. Additional studies are needed to assess the impact of repeated exposures to pathogens. Clinical studies are also needed to assess acquisition of pathogens during and after therapy with the antibiotics evaluated in the current study. In a previous investigation, it was demonstrated that piperacillin/tazobactam achieves sufficient concentrations in the intestinal tract of patients to inhibit *C. difficile* colonization during therapy [25]. Finally, we did not assess the impact of other host defense mechanisms such as gastric acid production on colonization resistance [1, 26].

## CONCLUSION

In conclusion, our findings demonstrate that several beta-lactam antibiotics recently developed for treatment of infections with resistant Gram-negative bacilli have the potential to promote colonization by healthcare-associated pathogens. Clinical studies are needed to examine the concentrations of these agents in the stool of patients and to assess the impact on the indigenous microbiota and the frequency of acquisition of colonization by healthcare-associated pathogens. Stewardship efforts are needed to ensure that these agents are used appropriately and for appropriate durations. Finally, there is a need to identify antibiotics that selectively target resistant Gram-negative bacilli without altering the indigenous microbiota that provide colonization resistance (eg, aztreonam, lolamicin) [1, 12].
